# Thermoregulation of Capsule Production by *Streptococcus pyogenes*

**DOI:** 10.1371/journal.pone.0037367

**Published:** 2012-05-17

**Authors:** Song Ok Kang, Jordan O. Wright, Rafael A. Tesorero, Hyunwoo Lee, Bernard Beall, Kyu Hong Cho

**Affiliations:** 1 Department of Microbiology, Southern Illinois University Carbondale, Carbondale, Illinois, United States of America; 2 Center for Pharmaceutical Biotechnology, University of Illinois, Chicago, Illinois, United States of America; 3 Streptococcus Laboratory, Centers for Disease Control and Prevention, Atlanta, Georgia, United States of America; Institut Pasteur, France

## Abstract

The capsule of *Streptococcus pyogenes* serves as an adhesin as well as an anti-phagocytic factor by binding to CD44 on keratinocytes of the pharyngeal mucosa and the skin, the main entry sites of the pathogen. We discovered that *S. pyogenes* HSC5 and MGAS315 strains are further thermoregulated for capsule production at a post-transcriptional level in addition to the transcriptional regulation by the CovRS two-component regulatory system. When the transcription of the *hasABC* capsular biosynthetic locus was de-repressed through mutation of the *covRS* system, the two strains, which have been used for pathogenesis studies in the laboratory, exhibited markedly increased capsule production at sub-body temperature. Employing transposon mutagenesis, we found that CvfA, a previously identified membrane-associated endoribonuclease, is required for the thermoregulation of capsule synthesis. The mutation of the *cvfA* gene conferred increased capsule production regardless of temperature. However, the amount of the capsule transcript was not changed by the mutation, indicating that a post-transcriptional regulator mediates between CvfA and thermoregulated capsule production. When we tested naturally occurring invasive mucoid strains, a high percentage (11/53, 21%) of the strains exhibited thermoregulated capsule production. As expected, the mucoid phenotype of these strains at sub-body temperature was due to mutations within the chromosomal *covRS* genes. Capsule thermoregulation that exhibits high capsule production at lower temperatures that occur on the skin or mucosal surface potentially confers better capability of adhesion and invasion when *S. pyogenes* penetrates the epithelial surface.

## Introduction

*S. pyogenes*, also known as Group A Streptococcus (GAS), is a Gram-positive human pathogen that causes a variety of diseases ranging from mild superficial infections such as impetigo and pharyngitis to life-threatening invasive or toxigenic diseases including myositis, necrotizing fasciitis, and streptococcal toxic shock syndrome. Immune-mediated post-streptococcal sequelae such as rheumatic fever and acute glomerulonephritis may develop following acute GAS infections such as pharyngitis. Diseases caused by *S. pyogenes* are a major public health concern both in developed and developing countries. A recent survey estimated that severe GAS diseases (post infectious sequelae and invasive infections) cause over a half million deaths each year [1]. More than 30 million cases of streptococcal pharyngitis and approximately 9,000–12,300 cases of invasive GAS disease occur each year in the U.S (see http://www.cdc.gov/abcs/reports-findings/surv-reports.html).

There is undoubtedly a complex interplay between the levels of expression of various bacterial virulence factors and environmental factors. Temperature shifts are important environmental signals that pathogens frequently encounter when initiating infection, since internal host temperatures are higher than environmental or external host surface temperatures. Thermoregulation of virulence factor levels potentially contributes to attachment to or invasion of host tissues through enhanced expression of adhesins or antiphagocytic factors and reduced expression of proteins normally targeted by the host immune responses [2], [3].

The *S. pyogenes* capsule is an adhesin involved in initial colonization at the lower temperatures typical of the mucosal surfaces of the throat or on the skin [4], [5], [6], and also serves as an antiphagocytic factor during invasive infections [7]. The capsule is composed of hyaluronic acid, the same polysaccharide found in human connective tissue, and binds to CD44 located on the surface of keratinocytes on the pharyngeal mucosa and the skin [5]. This activity of the *S. pyogenes* capsule promotes colonization and initiation of a new cycle of infection. The capsule biosynthetic operon is comprised of the *hasA*, *hasB*, and *hasC* genes, which encode hyaluronic acid synthetase, UDP-glucose dehydrogenase, and UDP-glucose pyrophosphorylase, respectively [8], [9], [10], [11]. The third gene in the operon *hasC*, however, is not required for capsule production due to the existence of another gene of UDP-glucose pyrophosphorylase in the genome [12], [13]. The transcription of the *hasABC* operon is under the control of a single upstream promoter [11], [14] that is regulated by the CovRS (also called CsrRS) two-component regulatory system, in which CovR is the response regulator and CovS is the sensor kinase [15]. The CovRS two-component system influences the expression of a large regulon that includes many virulence genes (reviewed in [16]). CovR primarily acts as a transcriptional repressor of genes that encode virulence factors, thus disruption of *covR* or *covS* affects the virulence of *S. pyogenes* in animal models (reviewed in [16]). Here we demonstrate that in a significant subset of invasive strains that contain defects in the *covRS* genes, the production of the GAS capsule adhesin and antiphagocytic factor is additionally controlled at a post-transcriptional level by environmental temperature. We speculate that through overproducing the capsule adhesin at sub-internal body temperatures these variants attach more effectively to epithelial cells, and that upon epithelial penetration, the thicker capsular layer potentially protects against the initial phagocytic attack leading to the systemic spread of invading GAS.

Previously we demonstrated that the GAS CvfA, a putative membrane-associated endoribonuclease, influences the expression of several virulence factors including M protein (cell wall-anchored protein exerting activities of adhesion and immune evasion), streptokinase (protease activating blood clot-cleaving enzyme plasmin), the CAMP factor (co-hemolysin), and SpeB (major secreted protease) in response to growth stages and nutritional stress [17]. Here we demonstrate that CvfA is also essential for thermoregulation of capsule synthesis.

## Materials and Methods

### Bacterial strains and media

Laboratory strains used in this study are listed in Table 1. *S. pyogenes* HSC5 [18] and MGAS315 [13] were used for most experiments and strain construction. HSC5 is a non-mucoid lab strain that has been studied for several streptococcal virulence factors and their regulations (selected references; [17], [18], [19], [20], [21], [22], [23], [24]). MGAS315 is a non-mucoid clinical strain isolated from a patient with streptococcal toxic shock syndrome, and its genome sequence is publically available [13]. *Escherichia coli* DH5α [25] or TOP10 (Invitrogen) was cultured in Luria-Bertani broth and was employed for plasmid construction. *S. pyogenes* was routinely cultured in Todd-Hewitt medium (BBL) supplemented with 0.2% yeast extract (Difco) at 37°C in sealed tubes without agitation. For solid media, Bacto agar (Difco) was added to a final concentration of 1.4% (w/v). Cultures on solid media were incubated under anaerobic conditions (<0.03% O_2_ and 15% CO_2_) employing a commercial gas generator (GasPak EZ Anaerobe container system). When appropriate, antibiotics were added to the media at the following concentrations unless specified; spectinomycin 100 µg/ml for *E. coli* and *S. pyogenes*; kanamycin 50 µg/ml for *E. coli* and 500 µg/ml for *S. pyogenes*; erythromycin 500 µg/ml for *E. coli* and 1 µg/ml for *S. pyogenes*; chloramphenicol 7 µg/ml for *E. coli* and 3 µg/ml for *S. pyogenes*.

**Table 1:** Bacterial strains and plasmids.

Strain/plasmid	Relevant genotype/description	Reference/source
**Strains**		
***E. coli***		
**DH5α**	*endA1 glnV*44 thi-1 *recA*1 *relA*1 *gyrA*96 *deoR nupG* ϕ80d*lacZ*ΔM15 Δ(*lacZYA-argF*)U169, *hsdR*17(r_K_^−^ m_K_^+^)	[25]
**TOP10**	*mcrA* Δ(*mrr-hsdRMS-mcrBC*) ϕ80*lacZ*ΔM15 Δ*lacX*74 *nupG recA*1 *araD*139 Δ(ara-leu)7697 *galE*15 *galK*16 *rpsL*(Str^R^) *endA*1	Invitrogen
***S. pyogenes***		
**HSC5**	Wild type, M14 serotype	[18]
**HSC5Spc**	Wild type control strain for pSPC18 insertion. pSPC18 is inserted into the HSC5 chromosome without disrupting any gene or operon.	[21]
**ΩCovR**	HSC5 strain with disrupted *covR* by a plasmid insertion. An internal part of *covR* (0.39 kb) was amplified by PCR, inserted into the suicide vector, pSPC18, and transferred into HSC5.	[20]
**ΩCovR(pCovRS)**	ΩCovR with the *covRS* genes expressed on pABG5.	This study
**CovRIFD**	HSC5 strain with in-frame deleted *covR*. This strain has a deletion from the leucine codon (L39) to valine codon (V183) in the gene of CovR (228 amino acid long). Refer to Materials and Methods.	This study
**CovRIFD:ΩSPy_2199**	CovRIFD with disrupted *SPy_2199* by the suicide vector pSPC18::'*SPy_2199'*	This study
**HasA-HA**	HSC5 strain producing HA (hemagglutinin) epitope - tagged HasA. The tagging was performed through homologous recombination using pCIV::*hasA*-HA.	This study
**CovRIFD:HasA-HAkm**	CovRIFD strain producing HA (hemagglutinin) epitope -tagged HasA. The tagging was performed through homologous recombination using pCIV::*hasA*-HA.	This study
**CovRIFD:HasA-HAspc**	CovRIFD strain producing HA (hemagglutinin) epitope -tagged HasA. The tagging was performed through homologous recombination using pSPC18:: '*hasA*-HA.	This study
**CovRIFD:TnCvfA1 & CovRIFD:TnCvfA2**	CovRIFD strains with transposon-generated CvfA^−^ mutation	This study
**CovRIFD:TnCvfA1:HasA-HA**	CovRIFD:TnCvfA1 strain producing HA (hemagglutinin) epitope -tagged HasA. The tagging was performed through homologous recombination using pSPC18:: '*hasA*-HA.	This study
**CovRIFD: TnCvfA1Comp**	CovRIFD:TnCvfA1 with pCvfA insertion into the chromosome. The disrupted *cvfA* in CovRIFD:TnCvfA1 was restored by inserting an intact copy of *cvfA*	This study
**ΩHasA**	HSC5 strain with disrupted *hasA*	[20]
**ΩHasA(pHasABC)**	ΩHasA with *hasABC* expressed on pABG5	This study
**CovRIFD:ΩHasA**	CovRIFD with disrupted *hasA*	This study
**CovRIFD:ΩHasA(pHasABC)**	CovRIFD:ΩHasA with *hasABC* expressed on pABG5	This study
**CovRIFD:TnCvfA1:ΩHasA**	CovRIFD:TnCvfA1 strain with disrupted *hasA*	This study
**CovRIFD:TnCvfA1:ΩHasA(pHasABC)**	CovRIFD:TnCvfA1:ΩHasA with *hasABC* expressed on pABG5	This study
**MGAS315**	Wild type, M3 phenotype	[13]
**MGAS315:ΩCovR**	MGAS315 strain with disrupted *covR*. The same method to create HSC5 ΩCovR was used.	This study
**Plasmids**		
**pSPC18**	pUC18-based streptococcal integration vector containing *aad9* (spectinomycin resistance gene from *Enterococcus faecalis*).	[28]
**pCIV2**	pUC18-based streptococcal integration vector containing *aphA3* (kanamycin resistance gene).	[77]
**pABG5**	pNZ12-based multi-copy *E. coli – S. pyogenes* shuttle vector. Streptococcal genes can be expressed under the *rofA* promoter.	[37], [78]
**pCovRS**	pABG5 containing the *covRS* genes (2.26-kbp) expressed under the *rofA* promoter. The *covRS* genes were amplified by PCR with the primers of 5covRSEcoRI (AAA*GAATTC*GAGGATAAGGGTTGGTATAA) and 3covRSHAPstI (AAA*CTGCAG*CTAAGCGTAATCTGGAACATCGTAGCTGGTACTCTCTTTAGACTGGGCCAAAGG) and inserted at the downstream of the *rofA* promoter on pABG5.	This study
**pSPC18::'** ***SPy_2199'***	pSPC18 containing a 0.63-kbp internal fragment of *spy_2199* amplified by PCR with the primers of 5KOSPyM3_1850BamHI (AAAA*GGATCC*CGCCCAGTATCAGCCTAAAG) and 3KOSPyM3_1850BamHI AAAA*GGATCC*CGTTTTAATGCGATGTTGACTG	this study
**pCIV2::** ***hasA*** **-HA**	pCIV2 containing a 1.4 kbp DNA fragment amplified by PCR with the primers of *hasA*compEcoRI-f (CG*GAATTC*TTAAAAATATTTCTATGACTAGTTGAC) and *hasA*compHAPstI-r (AA*CTGCAG*TTAAGCGTAATCTGGAACATCGTATGGGTATTTAAAAATAGTGACCTTTTTACGTG). The DNA fragment contains HA sequence (AGCGTAATCTGGAACATCGTATGGGTA) fused to the 3′ side of *hasA*.	this study
**pSPC18::'** ***hasA*** **-HA**	pSPC18 containing a 1.0 kbp DNA fragment amplified by PCR with the primers of 5*hasA*PstI (AA*CTGCAG*GAGTTCAAACACAGATGCAATAC) and *hasA*compHAPstI-r (AA*CTGCAG*TTAAGCGTAATCTGGAACATCGTATGGGTATTTAAAAATAGTGACCTTTTTACGTG). The DNA fragment contains HA sequence (AGCGTAATCTGGAACATCGTATGGGTA) fused to the 3′ side of *hasA*.	this study
**pCvfA**	pSPC18 containing an intact copy of *cvfA* (1.80 kbp) amplified by PCR with the primers of CvfAcompSphI-f (ACAT*GCATGC*GAAGCCTACATCATGGACGAC) and CvfAcompSphI-r **(** TTTT*GCATGC*CTACTTGGCATAATCAACCG **)**	this study
**pHasABC**	pABG5 containing the *hasABC* genes (3.76-kbp) expressed under the *rofA* promoter. The *hasABC* genes were amplified by PCR with the primers of 5hasABC-LIC (TACTTCCAATCCAATGAAAAGAAAGAGGTGTAATTGTGCC) and 3hasABC-LIC (TTATCCACTTCCAATGCTCTAGTAAAGTTGTATAACACAAAAC) and inserted at the downstream of the *rofA* promoter on pABG5.	This study

Restriction sites embedded into primer sequences are italicized.

### Manipulation of DNA

Plasmid DNA was isolated by standard techniques and used to transform *E. coli* by the method of Lederberg and Cohen [17]. Transformation of *S. pyogenes* was performed by electroporation as described previously [26]. Restriction endonucleases, ligases, and polymerases were used according to the recommendations of the manufacturers. Chromosomal DNA was purified from *S. pyogenes* using GenElute™ Bacterial Genomic DNA Kit (Sigma). When required, DNA fragments were purified using Mini Elute™ gel extraction kit (Qiagen) following agarose gel electrophoresis.

### Transposon mutagenesis

For transposon mutagenesis, TnΩKm2, a Tn*Spc* derivative containing a kanamycin resistance determinant, was employed. In TnΩKm2, the spectinomycin gene (*aad9*) in Tn*Spc* (for the genetic structure of this transposon, refer to Figure 1 in [23]) was replaced with ΩKm2, which is the kanamycin resistant determinant *aphA-3* flanked by a strong Rho-independent transcriptional terminator at both ends. The construction process of ΩKm2 was described elsewhere [27]. Construction of a library of transposon insertions was conducted as described elsewhere [23]. Transposon insertion sites were identified by direct sequencing of chromosomal DNA with a primer binding to a site within the transposon. Comparison to the genomic database (http://www.ncbi.nlm.nih.gov/BLAST/) was used to identify the site of a transposon insertion.

**Figure 1:**
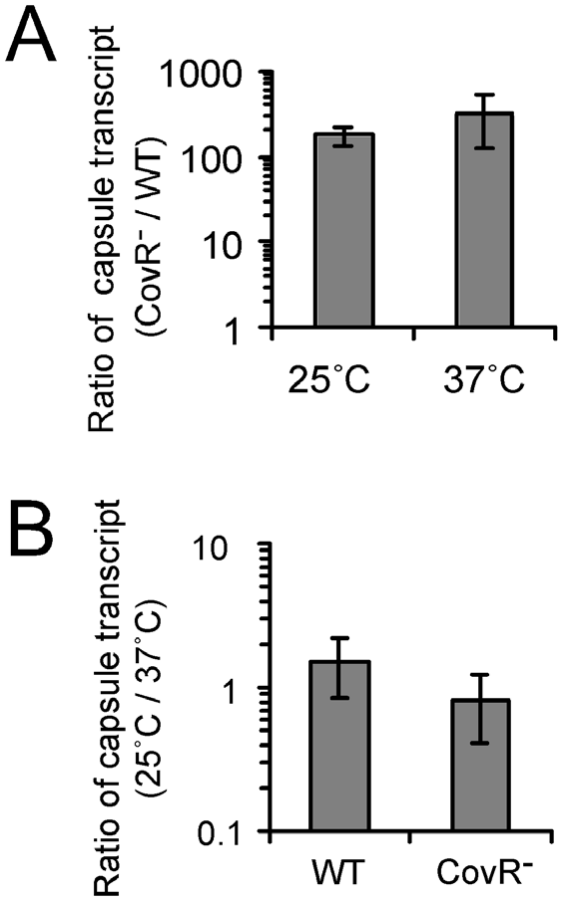
Capsule thermoregulation occurs at a post-transcriptional level. (A) The ratio of the capsule transcript quantity of the CovR null strain to that of the wild type. *S. pyogenes* cells were grown on THY agar plates at 37°C overnight (∼18 hrs) and the fully grown colonies were incubated for another six to eight hrs at each temperature (25°C or 37°C). After RNA was extracted from the colonies, the relative amounts of the capsule transcript between strains were measured through real time-RT (reverse transcriptase) PCR. Regardless of the incubation temperatures, the capsule transcript quantity in the CovR^−^ strain was more than 100 times higher than that in the wild type, confirming that the CovRS two-component system regulates the expression of the capsule transcript at the transcriptional level. (B) The ratio of the capsule transcript quantity at 25°C to that at 37°C. Regardless of the incubation temperatures, the capsule transcript quantity in both strains (the wild type and the CovR^−^ strain) was the same, indicating that thermoregulation of capsule production occurs at a post-transcriptional level. The following strains were used: wild type (WT), HSC5; CovR^−^ strain (CovR^−^), CovRIFD.

### Strain construction

#### Insertional disruption of the *covR* gene

The *covR* (also known as *csrR*, genomic locus SPyM3_0224) gene was disrupted by single crossover homologous insertion as follows: An internal region of *covR* was amplified by PCR, then inserted into the suicide vector pSPC18 [28]. The resultant plasmid was used to transform HSC5 or MGAS315. To ensure that strain phenotypes occurred specifically due to gene disruption and not to a non-specific effect of integration of pSPC18 into the chromosome, HSC5Spc (Table 1) was included in selected analyses as a wild type control strain for pSPC18 insertion [21].

#### In frame deletion of the *covR* gene

An in frame deletion in the *covR* gene was constructed as follows. The PCR primers, 5IFDcovRoutHind (AAAAAAGCTTTTGGGCAGTTTTGACAGAAG) and 3IFDcovRoutBam (AAAAGGATCCTTGTGATATCGCGCTCACTC), were used to amplify a region of the chromosome that includes the entire *covR* open reading frame using the high fidelity DNA polymerase Pfu (Invitrogen). The resulting fragment was inserted into the *E. coli*-streptococcal shuttle vector pJRS233 [29] after digestion with *Hind*III and *Bam*HI. The resulting plasmid (pJRS233::*covR*) was then used as the template in an inverse PCR reaction with primers 5IFDcovRinXma (AAAACCCGGGTTTGGAAATATGATGAAGCCG) and 3IFDcovRinXma (AAAACCCGGGACCCTTCACGACCATTGAC). This PCR product was digested with *Xma*I, then subjected to ligation and used to transform *E. coli*. The resulting plasmid, pJRS233::*covR*IFD, contained a 145 codon deletion derivative of *covR* with a unique *Xma*I site inserted at the deletion site (between codons 38 and 184 in *covR*). This allele was used to replace the wild type *covR* gene of HSC5.

#### *Cis*-complementation with the *cvfA* gene

Complementation of the transposon-inserted *cvfA* was performed with a method described elsewhere (For the scheme of complementation, refer to the Figure 1 in [17]). Briefly, the chromosomal region containing the *cvfA* gene and its putative promoter was amplified by PCR and inserted into the suicide vector pSPC18. The transposon-generated CvfA null strains were then transformed into strains producing CvfA by electroporating the *cvfA*-containing pSPC18. In the transformation process, the wild type *cvfA* allele in pSPC18 was inserted after the mutant allele, and this insertion was confirmed by PCR and restoration of the expression of SpeB, the major secreted protease, which is not produced from CvfA null strains [17].

#### *Trans*-complementation with *hasABC*

To express the *hasABC* transcript under a heterologous promoter, the entire *hasABC* genes including its ribosome-binding site was PCR-amplified and inserted downstream of the *rofA* promoter in the *E. coli – S. pyogenes* shuttle vector, pABG5. The *rofA* promoter was chosen because its transcription activity is not influenced by the CovRS two-component system [30] nor environment temperature [31]. For this construction, we employed ligase-independent cloning described elsewhere [32]. Briefly, the vector pABG5 was amplified with 5′ phosphorylated primers, pABG5EcoRIup-LIC (5′P-ATTGGATTGGAAGTACGAATTCAGTTCCTCACAATAATGG) and pABG5BanIdn-LIC (5′P- ATTGGAAGTGGATAACGGCACCGGGCAAAAAGCTG), and the insert, *hasABC*, was amplified with primers 5*hasABC*-LIC (TACTTCCAATCCAATGAAAAGAAAGAGGTGTAATTGTGCC) and 3*hasABC*-LIC (TTATCCACTTCCAATGCTCTAGTAAAGTTGTATAACACAAAAC). The high fidelity DNA polymerase Pfu (Invitrogen) was used for the PCR reaction. After purified with the Mini Elute™ PCR purification kit (Qiagen), each PCR-amplified product was treated with T4 DNA polymerase and dGTP (for vector) or dCTP (for insert) at 25°C for 30 min, then 75°C for 20 min. This T4 DNA polymerase treatment with one nucleotide generates single-stranded 5′ ends (ATTGGATTGGAAGTA and ATTGGAAGTGGATAA for the vector, TACTTCCAATCCAAT and TTATCCACTTCCAAT for the insert) due to its 3′ to 5′ exonuclease activity. The exonuclease activity stops when T4 DNA polymerase meets the same nucleotide in DNA as the nucleotide added to the reaction. After mixing the vector and the insert for 20 min at room temperature, 10 µl of the mixture was used to transform *E. coli*.

The details of each plasmid construct, the sequences of the primers used to amplify DNA segments and the transformed strains are listed in Table 1. Relevant strain constructs were confirmed by examining their phenotypes or PCR using appropriate primers.

#### Tagging HasA with the hemagglutinin (HA) epitope

To examine the expression level of the capsule gene product with Western blot, the first gene product of the capsule transcript, HasA, was tagged with a 9 residue hemagglutinin (HA) epitope (YPYDVPDYA) by single cross-over homologous insertion as follows: To fuse the HA epitope sequence (AGCGTAATCTGGAACATCGTATGGGTA) to the 3′ side of *hasA*, the *hasA* gene was amplified through PCR in which the 3′ primer contained the sequence of the HA epitope. The amplified product, the *hasA* gene tagged with the HA epitope sequence, was then inserted into a suicide vector, pCIV2 (kanamycin resistant) or pSPC18 (spectinomycin resistant). The pCIV2-based construct was used to transform HSC5 and CovRIFD to HasA-HA and CovRIFD:HasA-HA1 respectively, and the pSPC18-based plasmid was used to transform CovRIFD and CovRIFD:TnCvfA1 (kanamycin-resistant) to CovRIFD:HasA-HA2 and CovRIFD:TnCvfA1:HasA-HA.

### Quantitation of *S. pyogenes* capsule

To determine the amount of hyaluronic acid capsule produced by *S. pyogenes* on agar plates, a spectrophotometric assay using the cationic carbocyanine dye, 1-ethyl-2-[3-(1-ethylnaphtho-[1,2-d]thiazolin-2-ylidene)-2-methylpropenyl]naptho-[1,2-d]thiazolium bromide (Sigma Chemical Co., St. Louis, MO) was developed [33]. Overnight liquid culture of *S. pyogenes* grown in THY medium at 37°C was serially diluted with PBS buffer to allow plating less than 30 colonies on THY agar plates, which were incubated at 37°C overnight (18∼24 hrs) in anaerobic jars. These plates were further incubated at different temperatures (25°C, 30°C, or 37°C) for six to eight hrs to examine the influence of incubation temperature on capsule production. Afterwards, single colonies were taken from the agar plate with a microspatula (Fisher, Ca. # 21-401-10) and suspended by vortexing in 10 ml of sterile deionized water. After suspension, a portion of the solution was taken to determine the number of colony-forming units (CFUs) by plating after serial dilution. If necessary, the chains of *S. pyogenes* cells were broken by vigorous agitation or vortexing before plating. After precipitating the cells through centrifugation at 6,000× g for 10 min, the capsule-containing supernatant was mixed with an equal volume of capsule-staining reagent (20 mg of the cationic carbocyanine dye and 60 µl of glacial acetic acid in 50 ml of 50% formamide). The absorbance of the mixture was then measured at an OD of 640 and the amount of capsule was calculated using a standard curve generated with known concentrations of hyaluronic acid. The amount of capsule (femtogram per CFU) was calculated from the quantity of capsule. Capsule quantitation was performed in triplicate for each sample and at least twice per each strain.

### RNA isolation and real time RT-PCR

Reverse-transcriptase real-time RT-PCR with RNA isolated from *S. pyogenes* was carried out as previously described [19]. Quantitation of capsule transcript employed two primer pairs to separately target *hasA* and *hasB*. The sequences of primers used to examine the expression of *hasA* were ATGCTGCAACAGGACATTTG and TTAATGATTGAGCAGCACGC, which amplify a middle region (114 bps from 470^th^ to 583^rd^) of *hasA* (1188 bps), and those used to examine the *hasB* expression were TCCCCAAACGCTAATTGAAG and TTTACTGGGGACTCCTGCTC, which amplify a 3′ region (105 bps from 825^th^ to 929^th^) of *hasB* (1209 bps). As expected, the primer sets revealed the transcript amount of *hasA* was the same as that of *hasB*. Since these primers cover most parts of the transcript of *hasA* and *hasB* that are essential genes for capsule production, it is highly likely that *hasA* and *hasB* in the capsule transcript in the tested strains are intact. The gyrase subunit A gene (*gyrA*), whose expression was determined with the primers, AACAACTCAAACAGGTCGGG and CTCCTTCACGGCTAGATTCG, was chosen as an internal control to normalize the expression of the capsule transcript between samples. The *gyrA* gene was previously used in a study of *S. pyogenes* gene expression upon temperature shift [31], and we confirmed through quantitative RT-PCR that the expression level of the *gyrA* gene did not change at different temperatures ranging from 25°C–37°C. Data reported represent the mean derived from a minimum of 2 independent experiments each performed on a different day. Each data point was determined through at least duplicate testing.

### Examination of HasA expression level using Western blotting

The strains with HasA tagged with a 9 residue hemagglutinin (HA) peptide were grown on THY agar plates as described in the procedure for capsule quantitation. Colonies (∼100) were suspended in 1 ml PBS buffer prewarmed at 25°C or 37°C, lysed with a bead beater (FastPrep Instrument, MP biomedicals) at the speed of 6.0 for 30 sec twice, and boiled immediately for 10 min. This procedure was performed either in the lab (∼25°C) or in a 37°C culture room. The samples were then centrifuged for 5 min at the speed of 15,000× g to discard cell debris. The supernatant was transferred into a new tube, and the total protein amount was measured using the Bradford assay method. 10 µg of total protein was loaded onto an 8% SDS-PAGE gel and electrophoresed at 100 V for ∼2 hrs. The separated proteins on the gel were then transferred to a nitrocellulose membrane by electroblotting. After the transfer, colorimetric detection for the HA epitope on the membrane was performed by applying a rabbit anti-HA antibody (primary antibody, Invitrogen, 1∶1000 dilution), a goat anti-rabbit IgG antibody conjugated with HRP (horse radish peroxidase) (secondary antibody, Cell signaling, 1∶5000 dilution), and in turn the Bio-Rad Opti-4CN reagent. As an internal control for Western blotting, the expression level of SPy_2184, a membrane anchored protein (∼74 kDa) with a putative function of cyclic-di-AMP specific phosphodiesterase was determined. The expression of SPy_2184 is not controlled by the CovRS two-component system [30] or temperature (this study).

### Hyaluronidase activity assay

Hyaluronidase activity was determined by using an agar plate assay described elsewhere [34]. We used the *Streptococcus pneumoniae* 1121 Sm^r^ strain and its Δ*hyl* mutant as a hyaluronidase positive and a negative control strain, respectively [35].

## Results

### Temperature-dependent capsule production by CovR null strains

To investigate the thermoregulation of capsule production, *S. pyogenes* strains were plated (10–50 colonies per plate) on THY agar plates and incubated at 37°C anaerobically overnight (18–24 hrs). The fully-grown colonies were then incubated further for 6 to 8 hrs at each temperature tested (25°C, 30°C, or 37°C). The amount of hyaluronic acid capsule produced by each colony was then measured with a spectrophotometric assay.

CovR null strains of wild type *S. pyogenes* HSC5 and MGAS315 exhibited a high quantity of capsule only at temperatures below 37°C. Three CovR null strains were constructed for this assay: ΩCovR [20], a HSC5 CovR^−^CovS^−^ strain with insertionally disrupted *covR*; CovRIFD, HSC5 CovR^−^CovS^+^ strain with an in-frame deletion of *covR* (leucine-39 through valine-183); and MGAS315ΩCovR, an MGAS315 CovR^−^CovS^−^ strain with insertionally disrupted *covR*. Since *covS* is cotranscribed with *covR*, ΩCovR strains, whose *covR* was disrupted by a plasmid insertion produce neither CovR nor CovS [36]. All three CovR null strains produced 10 to 20 times more capsule at 25°C than at 37°C (Table 2). As expected, the wild type non-mucoid strains, HSC5 and MGAS315 expressed capsule at basal levels regardless of incubation temperature. When the capsule production of the CovR null strains incubated at 25°C, 30°C, or 37°C was compared, more capsule was produced at lower incubation temperatures (Table 2), indicating that capsule thermoregulation is gradual and is not regulated by an on/off mechanism.

**Table 2:** The amount of capsule produced by *S. pyogenes* strains at different temperatures.

Strain name	Description	Capsule produced (fg/cfu)%		
		at 37°C	at 30°C	at 25°C
HSC5	Wild type, M14 serotype	40.7±0.4	46.1±1.3	41.1±4.7
ΩCovR	HSC5 CovRS^−^ strain created by insertional disruption	37.0±0.3	276.1±10.9	755.7±73.7
CovRIFD	HSC5 CovR^−^ strain created by in-frame deletion	39.9±5.8	353.6±45.7	672.3±10.1
ΩCovR (pCovRS)	ΩCovR complemented with *covRS in trans*	42.3±4.0	49.8±0.3	42.0±5.9
CovRIFD:ΩSPy_2199	CovRIFD with disrupted *SPy_2199*, the immediate upstream gene of the capsule operon	29.9±12.0	ND*	656.1±39.6
MGAS315	Wild type, M3 serotype	85.5±0.7	ND*	84.2±2.5
MGAS31:ΩCovR	MGAS315 CovR^−^ strain created by insertional disruption	95.4±4.5	ND*	803.8±5.2

* ND: Not Determined.

% fg/cfu, femtogram per colony forming unit. Capsule quantitation was performed in triplicate for each sample and at least twice per each strain. The values shown here are average ± standard error.

The capsule amount produced by fully-grown *covR* null strain colonies under ambient air (20% O_2_ and 0.03% CO_2_) was not different from that under the anaerobic condition (<0.03% O_2_ and 15% CO_2_) (data not shown). Thus, the presence of oxygen or increased carbon dioxide concentration did not affect thermoregulation of capsule synthesis.

The open reading frame immediately upstream of the capsule operon, *SPy_2199*, a divergently transcribed gene encoding a putative protease, was disrupted in CovRIFD, and the resultant strain, CovRIFD ΩSPy_2199 showed the same phenotype of capsule thermoregulation as that of CovRIFD (Table 2), indicating no involvement of the upstream gene in capsule thermoregulation.

### Complementation of the *covR* null strain ΩCovR

Disruption of the *covR* gene also prevents the expression of the downstream gene *covS* since they are expressed under the same promoter. Thus, for *trans*-complementation of ΩCovR, both *covRS* genes (2.26 kb) were PCR-amplified and inserted downstream of the *rofA* promoter in the multi-copy plasmid pABG5 [37]. The promoter of *rofA*, the gene of a transcriptional activator (RofA, SPy_0124) regulating the expression of *prtF1* (fibronectin-binding protein 1), confers high expression on pABG5 [37]. When introduced into ΩCovR, the resulting plasmid pCovRS restored the wild-type phenotype (Table 2), confirming that the CovRS two-component system overrides capsule thermoregulation.

### Capsule thermoregulation occurs at a post-transcriptional level

To investigate the mechanism of capsule thermoregulation, we determined the relative amounts of the *hasABC* capsule transcript at different incubation temperatures using real-time RT-PCR. Regardless of incubation temperature, the level of the capsule transcript in CovRIFD was about 100 times more than that in the wild type, HSC5 (Figure 1A). This result confirmed the previous finding that the CovRS two-component system represses the capsule gene expression at the transcriptional level [15], [30], [38]. Interestingly, the level of the capsule transcript in CovRIFD grown at 25°C was almost the same as that in CovRIFD grown at 37°C (Figure 1B), even though the capsule amount of CovRIFD incubated at 25°C was about 17 times more than that incubated at 37°C (Table 2). This result suggests that the increased capsule amount of CovRIFD at the lower temperature resulted from a post-transcriptional event.

**Figure 2:**
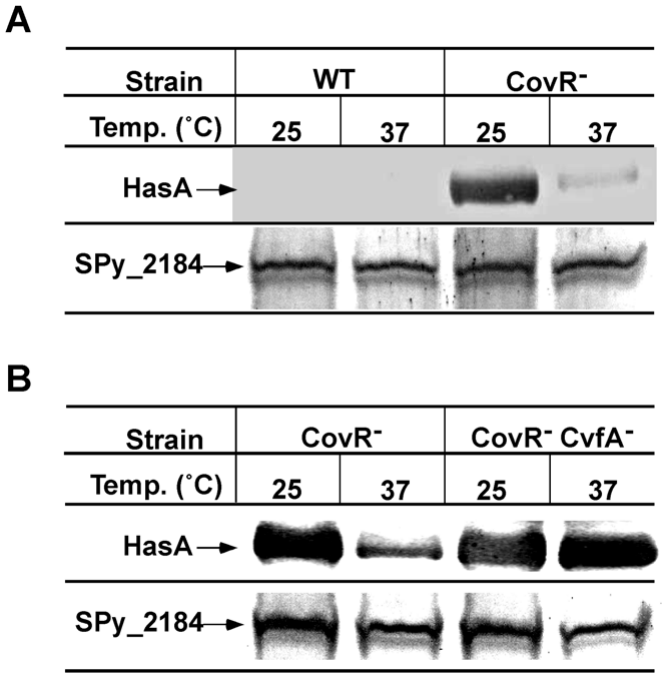
Capsule thermoregulation occurs at the production level of the capsule synthesis proteins. The first-translated capsule synthesis protein HasA was tagged with the HA (hemagglutinin) epitope through homologous recombination. The strains with HA-tagged HasA were grown on THY agar plates overnight. After being incubated further for another six to eight hours at 25°C or 37°C respectively, the colonies were collected and lysed with a bead beater. 10 µg of protein from each cell extract was loaded on 8% SDS-PAGE gel and electrophoresed. Western blotting against HA-tag or Spy_2184 (internal control) was then performed to examine the expression level of HasA according to temperature change. Figure A: The CovR^−^ strain produced ∼18 times more HasA at 25°C than 37°C. On the other hand, the wild type did not produce a detectable amount of HasA, regardless of the incubation temperatures. Figure B: The transposon-generated CvfA^−^ capsule overproducer at any temperature also overproduced HasA regardless of the incubation temperatures. Taken together with the result from Figure 1B, the thermoregulation of capsule production occurs at the production level of the capsule synthesis proteins. The following strains were used. Figure A: wild type (WT), HasA-HA; CovR^−^ strain (CovR^−^), CovRIFD:HasA-HAkm. Figure B: CovR^−^ strain (CovR^−^), CovRIFD:HasA-HAspc; CovR^−^ CvfA^−^ strain (CovR^−^ CvfA^−^); CovRIFD:TnCvfA1:HasA-HA.

We also analyzed the amount of a HasA-HA fusion product (HasA tagged with the hemagglutinin epitope) in strains grown at 25°C and 37°C through Western blotting using anti-HA antibodies (Figure 2). The CovRIFD strain produced the HasA protein 18 times higher at 25°C than at 37°C (compared using NIH ImageJ for densitometry), indicating that capsule thermoregulation occurs at a post-transcriptional level. As expected, the HasA protein was not detected from the wild type HSC5 at either temperature. The size of the HasA protein on the Western blot was the same as that calculated from the gene size and was the same between cells grown at 25°C and 37°C, revealing no obvious post-translational modification of HasA.

Taken together, capsule thermoregulation is governed at a post-transcriptional level, possibly at the translational level. Once the transcriptional repression by CovR is derepressed, capsule genes are transcribed at the same rate regardless of temperature but the production of HasA is apparently derepressed at 25°C relative to 37°C.

### HSC5 and MGAS315 do not exhibit extracellular hyaluronidase activity

Another factor that can influence the capsule production other than capsule synthesis rate is the degradation rate of hyaluronic acid capsule. Since some GAS strains (∼9% [39] or ∼23% [40]) produce an extracellular hyaluronidase that can degrade the hyaluronic acid capsule [40], [41], we examined if the strains HSC5 and MGAS315 produce the functional extracellular hyaluronidase HylA. All the GAS strains sequenced so far contain the sequence of *hylA*. However, the majority of GAS strains, which do not exhibit extracellular HylA activity, have mutations in *hylA* [39]. The strains we used also have mutations in *hylA*, compared to the full-length *hylA* gene of MGAS10750 (serotype M4), indicating that our strains do not produce functional HylA. The *hylA* gene of HSC5 (serotype M14) is terminated early by a nonsense mutation (Q806 to Ochre stop codon) resulting in a 63-amino acid truncation. The *hylA* gene of MGAS315 (serotype M3) has an inframe deletion resulting in a 61 amino acid deletion near the N-terminus of HylA. These mutations have been reported previously to produce non-functional HylA [39]. Consistent with this sequencing analysis, both our wild type strains and their CovR^−^ strains did not exhibit extracellular hyaluronidase activity either at 25°C or 37°C in a plate diffusion assay described elsewhere [34] (unpublished data).

### Occurrence of the mucoid phenotype among invasive GAS

The CDC *Streptococcus* Laboratory routinely notes the presence or absence of the highly mucoid phenotype among the invasive GAS received for characterization from Active Bacterial Core surveillance (ABCs). Among 2850 invasive isolates collected during 2007–2010 surveillance in 10 states, 59 isolates (2.1%, from 7 different states) displayed obvious excess production of capsule on sheep blood agar (SBA) plates. Type *emm1* (subtype *emm1.0*) represented 29 (49.2%) of these isolates, with types *emm6*, *emm11*, and *emm118* accounting for 5 isolates each. Types *emm3* and *emm89* each accounted for 4 isolates. Type *emm1* was the most common invasive type overall during 2007–2010, accounting for >23% of the total isolates, while the other 5 *emm* types accounting for 4–5 mucoid isolates occurred within 2.1%–7% of total invasive isolates.

### Capsule thermoregulation occurs among invasive clinical isolates of *S. pyogenes*

To examine the presence of capsule thermoregulation in clinical isolates, we tested 115 invasive isolates that were obtained through the CDC's Emerging Infections Program/Active Bacterial Core surveillance (ABCs) during 2007–2010. Among these 115 isolates, 53 isolates were mucoid on THY agar plates at 25°C and/or 37°C. Most of these mucoid strains had already exhibited mucoid phenotype in the CDC's screening using sheep blood agar plates. Among the 53 mucoid isolates, 11 (21%), representing 5 different *emm* types, demonstrated capsule thermoregulation in that they were observed to produce more capsule at the lower temperature (Table 3). The lab strains, HSC5 and MGAS315, exhibited capsule thermoregulation only when CovR-mediated transcriptional repression was released by mutations of the *covRS* genes. Since mutations in *covRS* have been reported to be frequently observed in invasive clinical isolates [42], [43], we tested if the eleven CDC strains that exhibited capsule thermoregulation contained mutations in the *covRS* genes. First, we introduced the pCovRS plasmid to the eleven CDC strains and examined the phenotype for capsule thermoregulation. The introduction of pCovRS abolished capsule production of all the capsule-thermoregulated invasive isolates at either temperature (Table 3). This result indicates that the introduction of pCovRS complemented defective CovRS-mediated repression of the *hasABC* operon, so both or either CovR or CovS would be nonfunctional in those clinical isolates. For further confirmation of the complementation analysis, we sequenced the *covRS* region of the 11 isolates. As expected, all 11 isolates contained mutation within *covR* or *covS* (Table 4). Interestingly, mutation in *covS* was dominant in these isolates; eight isolates have mutations in *covS* and only three isolates have mutations in *covR*. Most mutations (7 out of 8) in *covS* were frame shift mutations causing early termination of translation, and these frame shift mutations were caused by deletion of redundant sequences (isolates 2, 17, 36, 37, 53) or duplication of a sequence (isolates 9 and 47). The most frequent deletion was deletion of a thymine out of a 7 consecutive thymine sequence. On the other hand, all of the three mutations in *covR* were missense mutations, of which two were previously reported to confer the mucoid phenotype [42], [43].

**Table 3:** Capsule production by mucoid invasive *S. pyogenes* strains at different temperatures.

CDC lab isolate ID#	Source	*emm* subtype	Mucoid at 37°C*	Mucoid at 25°C*	Capsule thermo-regulation	pCovRS complementation@	
						Mucoidity at 37°C*	Mucoidity at 25°C*
1**	Blood	*emm*1.0	**+**	**+**	No		
2	Blood	*emm*3.1	**−**	**+++**	**Yes**	**−**	**−**
3**	Blood	*emm*11.0	**−**	**+++**	**Yes**	**−**	**−**
4	Blood	*emm*18.0	**+++**	**+++**	No	**+**	**+**
5	Blood	*emm*95.0	**+**	**+**	No		
6**	Blood	*emm*118.0	**++**	**++**	No		
7	Cerebral Spinal Fluid	*emm*122.2	**+++**	**+++**	No	**−**	**−**
8	Blood	stns554.0%	**++**	**++**	No		
9**	Blood	*emm*89.0	**+**	**++**	**Yes**	**−**	**−**
10**	Fascia Tissue	*emm*1.0	**+**	**+**	No		
11**	Blood	*emm*11.0	**+**	**+**	No		
12**	Blood	*emm*1.0	**+**	**++**	**Yes**	**−**	**−**
13**	Blood	*emm*1.0	**+**	**+**	No	**−**	**−**
14**	Blood	*emm*1.0	**++**	**++**	No		
15**	Blood	*emm*1.0	**+**	**+**	No		
16**	Pleural Fluid	*emm*6.0	**++**	**++**	No		
17**	Blood	*emm*89.0	**++**	**+++**	**Yes**	**−**	**−**
18**	Blood	*emm*1.0	**+**	**+**	No		
19**	Blood	*emm*1.0	**+**	**+**	No		
20**	Blood	*emm*118.0	**+++**	**+++**	No	**−**	**−**
21**	Blood	*emm*1.0	**++**	**++**	No		
22**	Blood	*emm*1.0	**+**	**+**	No		
23**	Blood	*emm*6.4	**++**	**++**	No		
24**	Blood	*emm*6.74	**++**	**++**	No		
25**	Blood	*emm*1.0	**+**	**+**	No		
26**	Blood	*emm*11.0	**++**	**++**	No		
27**	Blood	*emm*1.0	**+**	**+**	No		
28**	Blood	*emm*1.0	**+**	**+**	No		
29**	Blood	*emm*1.0	**++**	**++**	No		
30**	Blood	*emm*83.1	**+++**	**+++**	No	**+**	**+**
31**	Blood	*emm*3.1	**+**	**+**	No		
32**	Blood	*emm*11.0	**+**	**+**	No		
33**	Blood	*emm*1.0	**++**	**++**	No		
34**	Blood	*emm*1.0	**+**	**+**	No		
35**	Blood	st4722.0%	**+**	**+**	No		
36**	Blood	*emm*1.0	**+**	**+++**	**Yes**	**−**	**−**
37**	Blood	*emm*1.0	**++**	**+++**	**Yes**	**−**	**−**
38**	Blood	stPA57.3%	**+++**	**+++**	No	**++**	**++**
39**	Blood	*emm*44.0	**+++**	**+++**	No	**−**	**−**
40**	Blood	*emm*89.0	**++**	**++**	No		
41**	Blood	*emm*1.0	**++**	**++**	No		
42**	Blood	*emm*3.1	**++**	**++**	No		
43**	Blood	*emm*1.0	**++**	**++**	No		
44**	Blood	*emm*1.0	**+++**	**+++**	No	**−**	**−**
45**	Blood	*emm*1.0	**++**	**++**	No		
46**	Blood	*emm*1.0	**++**	**+++**	**Yes**	**−**	**−**
47**	Blood	*emm*3.1	**+**	**+++**	**Yes**	**−**	**−**
48**	Blood	*emm*18.0	**+++**	**+++**	No	**+**	**+**
49**	Blood	*emm*59.0	**+++**	**+++**	No	**−**	**−**
50**	Pleural Fluid	*emm*118.0	**++**	**+++**	**Yes**	**−**	**−**
51**	Blood	*emm*118.0	**+++**	**+++**	No	**−**	**−**
52**	Blood	*emm*118.0	**+++**	**+++**	No	**−**	**−**
53**	Blood	*emm*11.0	**+**	**+++**	**Yes**	**−**	**−**

# Isolates were recovered through Active Bacterial Core surveillance for *S. pyogenes* during 2007–2010 in the United States. Refer to surveillance reports at http://www.cdc.gov/abcs/reports-findings/surv-reports.html.

% New *emm* subtypes that have not yet been provided *emm* designations. All *emm* designations are shown at the downloadable database ftp://ftp.cdc.gov/pub/infectious_diseases/biotech/tsemm/.

* Mucoidity assay was performed with colonies grown on THY (Todd Hewitt Yeast) agar plates.

− : Basal level capsule production (less than 100 fg/cfu), not observable with visual inspection such as HSC5 or MGAS315 wild type.

+ : Capsule production of 100–300 fg/cfu, barely detectable with visual inspection.

++ : Capsule production of 300–500 fg/cfu, clearly detectable with visual inspection but less amount of capsule production than HSC5 CovRS null mutants incubated at 25°C.

+++ : Capsule production of more than 500 fg/cfu, clearly detectable with visual observation, almost the same amount of capsule production as HSC5 CovRS null mutants incubated at 25°C.

** These strains were preselected as being mucoid on blood agar plates by CDC.

@ Blank cells: Values were not determined.

**Table 4:** Alterations in the *covRS* sequence of strains exhibiting capsule thermoregulation.#

CDC lab isolate ID	Mutated gene	Type of mutation*	Consequence of mutation%	Reference
2	*covS*	Frame shift (deletion of nt 77)^a^	Early termination after aa residue 35	[42], [43]
3	*covR*	Missense mutation	M86V	[42]
9	*covS*	Frame shift (insertion of 11 bps between nt 82 and nt 83)^b^	Early termination after aa residue 39	[43]
12	*covS*	Missense mutation	T35I, V57G	This study
17	*covS*	Frame shift (deletion of nt 77)^a^	Early termination after aa residue 35	[42], [43]
36	*covS*	Frame shift (deletion of nt 77)^a^	Early termination after aa residue 35	[42], [43]
37	*covS*	Frame shift (deletion between nt 1205 and nt 1211)^c^	Early termination after aa residue 407	[43]
46	*covR*	Missense mutation	A81T	This study
47	*covS*	Frame shift (insertion of 11 bps between nt 88 and nt 89)^d^	Early termination after aa residue 39	[43]
50	*covR*	Missense mutation	R94C	[42], [43]
53	*covS*	Frame shift (deletion of nt 77)^a^	Early termination after aa residue 35	[42], [43]

# The CovRS sequences of CDC lab isolates were compared to the CovRS sequences of 11 genome-sequenced strains publically available at the NCBI website, http://www.ncbi.nlm.nih.gov/genomes/lproks.cgi.

* Nucleotide sequence level; nt, nucleotide.

% Protein sequence level; aa, amino acid.

a Deletion of a thymine out of 7 consecutive thymines.

b Duplication of 11 bps of TCTGCATTTTC.

c Deletion of 5 bps of AAAGA out of AAAGAAAAGA.

d Duplication of 11 bps of TTTTCTCTGCC.

Another eleven CDC lab isolates (isolate # 4, 7, 20, 30, 38, 39, 44, 48, 49, 51, 52) among the 53 mucoid invasive isolates were highly mucoid regardless of incubation temperature, indicating that both the transcriptional repression by the CovRS system and capsule thermoregulation do not function in these strains. As expected, complementation with pCovRS reduced the mucoidity of these strains at the same degree at both incubation temperatures. Several isolates (isolate # 4, 30, 38, 48) exhibited some mucoidity after pCovRS complementation, even though the degree of mucoidity decreased (Table 3). *S. pyogenes* mucoidity is dependent on not only the functionality of the CovRS system but also the sequence of the capsule promoter region where CovR binds [44]. Thus, the CovR binding sites in the capsule promoter region of the strains exhibiting some mucoidity after the complementation with pCovRS might have less affinity for CovR.

### CvfA, an endoribonuclease, is required for capsule thermoregulation

**Figure 3:**
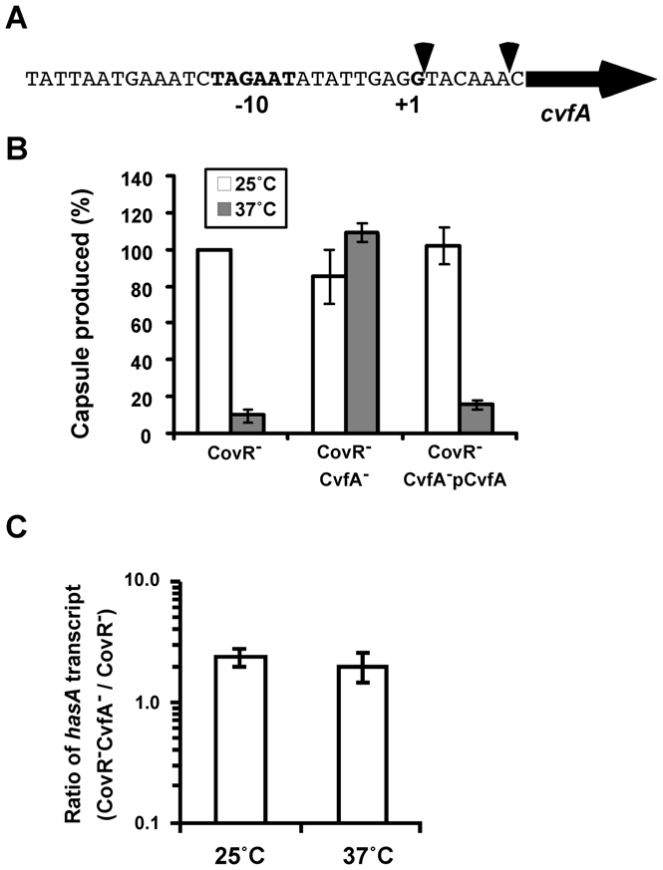
CvfA is required for the thermoregulation of capsule production. A) Transposon insertion sites in the transposon-generated CvfA^−^ mutants. The nucleotide sequence in front of the start codon of *cvfA* is shown. The horizontal arrow represents 5′- side of the *cvfA* gene. The putative −10 promoter element and transcription start site (+1) are indicated in bold. Each transposon insertion site in the transposon-generated CvfA^−^ strains was shown with a vertical arrowhead: transposon insertion between G and T in the CovRIFD:TnCvfA1 strain and between A and C in the CovRIFD:TnCvfA2 strain. These transposon insertions eliminated thermoregulation of capsule production. B) The transposon-generated CvfA^−^ mutant regained the phenotype of capsule thermoregulation upon *cis*-complementation. The transposon insertions that prevent the expression of *cvfA* abolished the capsule thermoregulation of the CovR*^−^* background strain; the transposon-generated CvfA^−^ strain overproduced capsule regardless of the incubation temperatures. The introduction of an intact copy of *cvfA* into the chromosome of the transposon-generated CvfA^−^ strain (*cis*-complementation) restored capsule thermoregulation. C) The transposon-generated CvfA^−^ mutation did not influence the capsule gene transcription even at 25°C. The transposon-generated CvfA mutant was grown on THY agar plates at 37°C overnight (∼18 hrs) and the fully-grown colonies were incubated for another six to eight hrs at each temperature (25°C or 37°C). The quantity of the capsule transcript in the strain was then measured with real time-RT (reverse transcriptase) PCR and compared to that in the CovR^−^ background strain. The following strains were used: CovR^−^ strain (CovR^−^), CovRIFD; CvfA^−^ CovR^−^ strain (CovR^−^CvfA^−^), CovRIFD:TnCvfA1; CvfA complemented strain of CovRIFD:TnCvfA1 (CovR^−^CvfA^−^pCvfA), CovRIFD:TnCvfA1Comp.

In order to identify genes or chromosomal loci that influence capsule thermoregulation, transposon mutagenesis was performed using a Tn*4001* derivative containing the kanamycin resistance gene, *aphA-3*. The transposon has a strong Rho-independent transcriptional terminator at both ends of *aphA-3*, so no leakage of transcription from the transposon occurs. The *covR* in-frame deletion strain, CovRIFD, was used as the capsule-thermoregulated background strain for the transposon mutagenesis. Among transposon-generated mutants, eight strains produced no capsule at either 25°C or 37°C. DNA sequencing to identify the transposon insertion site revealed that each of these strains that lacked capsule production at either temperature contained a transposon insertion within the *hasABC* operon, confirming that the over-produced capsular material from the CovR null strains at 25°C was hyaluronic acid. Among transposon-generated mutants, two strains in which the transcription of the *cvfA* gene *(Spy_1633)* was disrupted by an insertion of the transposon overproduced capsule at both temperatures. In these strains, CovRIFD:TnCvfA1 and CovRIFD:TnCvfA2 (*covR* in-frame deletion strain with a transposon insertion preventing *cvfA* transcription), the transposon was inserted in the location just upstream of the start codon of *cvfA* (Figure 3 A). Capsule amount from these strains increased more than 10-fold at 37°C, compared to that of CovRIFD (Figure 3 B), while capsule gene transcription within these transposon-generated capsule overproducers changed very little. The capsule transcript ratio of CovRIFD:TnCvfA1 to CovRIFD was 2.37±0.41 (mean ± standard deviation) at 25°C and 2.01±0.55 at 37°C (Figure 3 C).

Previous Western blot analysis exhibited that the overproduced capsule amount by CovRIFD at 25°C was directly correlated with the amount of the capsule synthesis proteins. As expected, the capsule amount produced by the transposon-generated capsule overproducers also directly correlated with the amount of the capsule synthesis proteins; the mutant strain CovRIFD:TnCvfA1 overproduced HasA regardless of the incubation temperature, so it produced higher amounts of HasA than the background strain CovRIFD at 37°C (Figure 2 B). These results are consistent with capsule thermoregulation occurring at a post-transcriptional level.

Our previous work indicated that the *cvfA* gene is monocistronic and its disruption does not affect the expression of downstream genes [17]. To confirm that the lack of *cvfA* expression due to the transposon insertion was solely responsible for the phenotype of capsule overproduction, the *cvfA* gene and promoter region were amplified, inserted into the suicide vector pSPC18 and transferred into the transposon-generated capsule overproducer, CovRIFD:TnCvfA1, as previously described [17]. This *cis*-complemented strain, CovRIFD:TnCvfA1Comp, exhibited capsule thermoregulation identical to the parental strain (Figure 3 B), verifying that *cvfA* is required for thermoregulation of capsule synthesis.

### Complementation analysis with the capsule genes expressed *in trans* under a heterologous promoter confirms that thermoregulation occurs at the post-transcriptional level where CvfA is required

**Figure 4:**
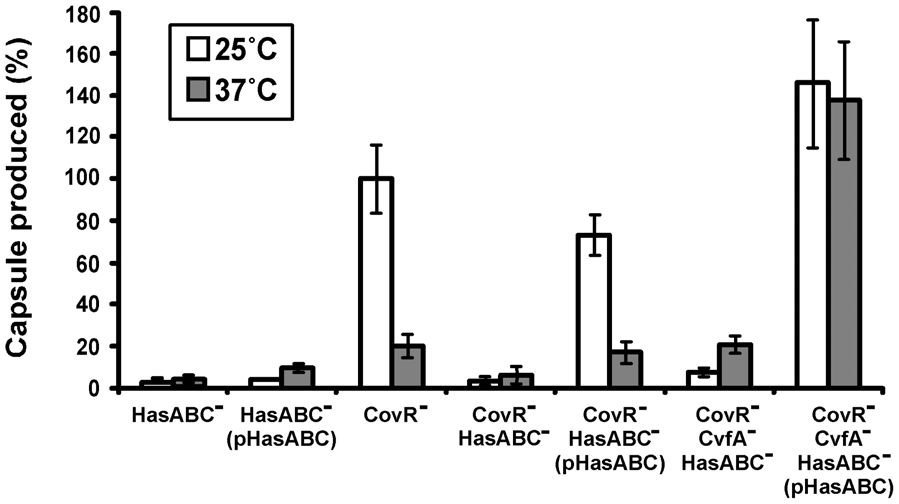
Expression of the capsule genes *in trans* under a heterologous promoter also exhibits capsule thermoregulation. The *hasABC* genes were expressed *in trans* under the *rofA* promoter on pHasABC and transferred to *S. pyogenes* HSC5 strains whose expression of chromosomal capsule genes was blocked by disrupting the first transcribed capsule gene, *hasA*. Those complemented strains with pHasABC exhibited the same phenotype of capsule thermoregulation as the strains whose capsule genes were expressed under the native promoter on the chromosome. The following strains were used: CovR^−^ strain (CovR^−^), CovRIFD; chromosomal capsule gene knock-out strain (HasABC^−^), ΩHasA; *in trans* capsule gene complemented strain (HasABC^−^(pHasABC)), ΩHasA(pHasABC); CovR^−^ HasABC^−^ strain (CovR^−^ HasABC^−^); CovRIFD:ΩHasA; CovR^−^ HasABC^−^ strain complemented with pHasABC (CovR^−^ HasABC^−^ (pHasABC)), CovRIFD:ΩHasA(pHasABC); CovR^−^ CvfA^−^ HasABC^−^ strain (CvfA^−^ CovR^−^ HasABC^−^), CovRIFD:TnCvfA1:ΩHasA; CovR^−^ CvfA^−^ HasABC^−^ strain complemented with pHasABC (CvfA^−^ CovR^−^ HasABC^−^ (pHasABC)), CovRIFD:TnCvfA1:ΩHasA(pHasABC).

**Figure 5:**
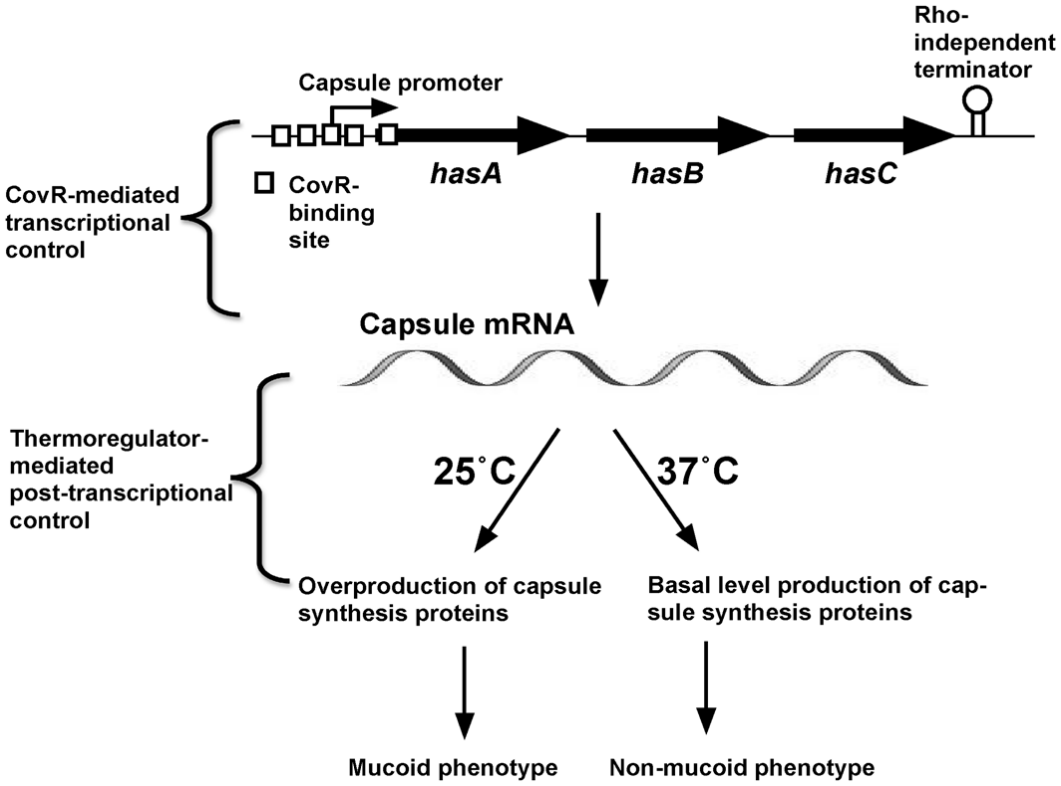
Regulation of capsule production by *S. pyogenes* strains, HSC5 and MGAS315. Capsule production by the non-mucoid *S. pyogenes* strains, HSC5 and MGAS315, is regulated both at the transcriptional and post-transcriptional level. Phosphorylated CovR binds to its binding sites in the capsule promoter region, and represses *hasABC* transcription [45]. A thermoregulatory system regulates the production of capsule synthesis proteins at a post-transcriptional level. Capsule production occurs only when CovR is not bound to the *hasABC* promoter region and environmental temperature is lower than 37°C.

*S. pyogenes* strains were transformed to express the capsule genes *in trans* under the *rofA* promoter and the phenotype was examined for capsule thermoregulation (Figure 4). Expression of the chromosomal capsule genes was blocked by disrupting the first gene of the capsule operon *hasA* in order to examine only the influence of ectopically expressed *hasABC*. The capsule genes under the *rofA* promoter were not expressed in the wild type strain HSC5. One of the five CovR-binding sites in the capsule promoter region resides at the end of the 5′ side of *hasA* (refer to Figure 5), and that CovR binding site has the strongest transcription inhibitory activity [45]. As expected, when the *covR* gene was deleted, the strain CovRIFD:ΩHasA(pHasABC), exhibited capsule thermoregulation, indicating that the native capsule gene promoter is not required for capsule thermoregulation. This result also confirms that capsule thermoregulation occurs at a post-transcriptional level. When the CvfA^−^ capsule overproducer was transformed with pHasABC, the strain CovRIFD:TnCvfA1:ΩHasA(pHasABC) overproduced capsule regardless of incubation temperature. Thus, CvfA is required for capsule thermoregulation even when the capsule genes are expressed under a heterologous promoter *in trans*.

## Discussion

*S. pyogenes* strains have been classified as non-mucoid or mucoid depending upon capsule production under laboratory growth conditions (mostly grown on rich media at 37°C anaerobically). While some strains do not produce capsule under laboratory growth conditions, genomes of all *S. pyogenes* strains contain the intact *hasABC* capsular biosynthetic operon, suggesting that certain factors or conditions for capsule production by non-mucoid strains might be missing. It is known that CovR, the response regulator of the CovRS two-component system, binds to the promoter region of the capsule operon and represses the transcription of capsule genes, consistent with the observation that spontaneous mutations within the two-component system *covRS* genes confer the mucoid phenotype [42]. The influence of environmental temperature upon capsule production by CovRS defective strains has not been previously described.

As expected, we found in our lab strains that when *covR* was inactivated, the amount of capsule gene transcript dramatically increased (Figure 1). Still, only when these CovR null strains were grown at sub-body temperature was a very large increase in capsule production observed (Table 2). Our study indicates that while the transcription of capsule genes is controlled in a temperature-independent manner by the CovRS two-component system, a post-transcriptional regulation of capsule production occurs in a temperature-dependent manner (Figure 5).

Why does *S. pyogenes* overproduce capsule at sub-body temperature? The capsule is composed of hyaluronic acid polymer identical to that found in human connective tissue, and binds to CD44 on keratinocytes in the pharyngeal mucosa and the skin [5]. Major functions of increased capsule production may be to protect from desiccation and to increase the efficiency of initial attachment to new host epithelial cells, thus promoting the spread of the organism amongst the human population. Such roles are consistent with *S. pyogenes* optimally producing the capsule outside or on the surface of the host epithelial layers, where the temperature is usually lower than the body temperature. Consistent with this notion, our results show that in strains thermoregulated for capsule synthesis, capsule production decreases when temperature increases (Table 2). Nonetheless, it is likely that the enhanced capsular layer serves to protect strains upon initiation of systemic infection [46], [47], [48]. Possibly, soon after invasion through the epithelial layer, enhanced capsular expression may not be required to evade phagocytosis due to expression of M protein and/or other antiphagocytic factors including streptococcal C5a peptidase. Husmann et al. showed that capsule is required for pharyngeal colonization and increased survival in the lower respiratory tract resulting in pneumonia in respiratory infection mouse models [4]. However, capsule plays a much smaller role in resistance to phagocytosis than M protein in human blood [4]. These results agree with our hypothesis that capsule increases invasion (penetration) by enhancing adherence to keratinocytes at lower temperature and capsule might not be required for evading phagocytosis due to the presence of other antiphagocytic factors.

Paradoxically, *S. pyogenes* has been known to exhibit an extracellular hyaluronidase activity that cleaves the 1.4 ß-linkage between N-acetylglucosamine and D-glucuronic acid, the repeating subunit of hyaluronic acid capsule. However, contrary to popular belief, only a small fraction (∼9% [39] and ∼23% [40]) of clinical strains exhibit the extracellular hyaluronidase activity. Most invasive strains including M1 and M3 serotypes do not exhibit the extracellular hyaluronidase activity, stressing the importance of capsule in invasive *S. pyogenes* strains [39]. The genome of *S. pyogenes* contains several hyaluronidase genes, one in the GAS chromosome and the others in prophage genes. Among these hyaluronidases, only the product of *hylA*, the hyaluronidase gene encoded in the GAS chromosome, is attributable to the extracellular hyaluronidase activity [40], [41]. Other hyaluronidases encoded in phage genes appeared not to be exported into the extracellular milieu [40], suggesting that the role of these prophages' hyaluronidases is to help the prophages escape from their encapsulated host. The majority *S. pyogenes* strains, which do not exhibit the extracellular hyaluronidase activity, possess mutations in *hylA*. Our lab strains, HSC5 and MGAS315, do not produce the functional extracellular hyaluronidase since *hylA* in both strains have mutations that have been reported previously to produce non-functional HylA [39]. Thus, the extracellular hyaluronidase HylA, which can degrade the hyaluronic acid capsule, does not affect our interpretation of the post-transcriptional capsule thermoregulation. Another line of evidence that HylA does not play a major role in capsule thermoregulation is the Western blot analysis (Figure 2), which exhibits a direct correlation between the amount of the capsule synthesis proteins and the amount of capsule produced. Taken together, these results indicate that capsule thermoregulation occurs directly by the regulation of the production of the capsule gene products, not by indirect mechanism such as the regulation of the extracellular hyaluronidase, HylA.

We tested invasive *S. pyogenes* isolates provided by the CDC for capsule thermoregulation and found that capsule thermoregulation is a common feature, with 21% of the mucoid isolates displaying the capsule thermoregulation phenotype. As expected, those isolates displaying capsule thermoregulation contained mutations within the *covRS* genes. This result confirms previous reports that CovRS mutation is frequent among clinical invasive strains [42], [43]. When Ikebe *et al.* examined a panel of clinical isolates from cases of invasive human infections, nearly half of them (76 out of 164 isolates) contained mutations in CovR or CovS [43]. Also, spontaneous emergence of CovRS^−^ mutants was observed from a murine skin infection model, where it was demonstrated that these mutations enhanced strain virulence and increased severity of infection [42]. These findings indicate that mutations within *covRS* are selected *in vivo* due to the better survival through increased virulence factor expression. Unlike activators for virulence gene expression, mutations in the CovRS two-component system, a repressor system, increases the expression of many virulence factors. Our discovery of capsule thermoregulation among naturally occurring virulent *covRS* mutation strains is another potentially important aspect of the virulence triggering mechanism in this organism [49], especially by the *emm1* strains that we found to over-represent mucoid invasive isolates.

Even though only *covRS* mutation strains display capsule thermoregulation under the assay condition we applied, wild type strains with functional CovRS such as HSC5 and MGAS315 may be capable of exerting capsule thermoregulation in natural infection locations such as on the skin or the mucosal surface. In many bacterial species, response to environmental stress is mediated by secondary sigma factors. *S. pyogenes*, however, does not contain stress-responsive sigma factors, and the role of stress-response in GAS appears to be largely mediated by the CovRS two-component system which is required for growth under adverse conditions such as elevated temperature [36], low pH [36], high salt concentration [36], low magnesium [50], iron starvation [51], and the presence of the cationic human antimicrobial peptide LL-37 [51], [52]. In these conditions, CovR is dephosphorylated and the transcriptional repression by CovR is released. The epithelial surface of infection sites could provide the stress conditions that result in CovR dephosphorylation, including the lack of certain nutrients (magnesium and iron for example), acidic pH [53], and the presence of antimicrobial peptides.

Several mechanisms of thermoregulation have been discovered in bacterial pathogens, involving DNA supercoiling, mRNA secondary structure, and regulatory proteins. The degree of DNA supercoiling, which modulates the binding of regulatory proteins, is associated with thermoregulation [54], [55], [56], [57], [58], [59]. Previous studies suggest that histone-like proteins such as H-NS modulate gene expression based upon the degree of DNA supercoiling [56], [60], [61], [62], [63]. Changes in mRNA secondary structure, “riboswitches”, can confer thermoregulation. For example, the secondary structures of the Shine-Dalgarno region of *lcrF* mRNA in *Yersinia pestis* [64] and *prfA* in *Listeria monocytogenes* [65] change in response to temperature shift. At body temperature (37°C), a loop structure of the mRNAs of *lcrF* and *prfA* melts, which frees the Shine-Dalgarno region from their secondary structures and allows translation to proceed. Some DNA-binding regulators exhibit differential affinity to their DNA binding sites at different temperatures. This type of thermoregulation requires DNA-binding regulators to be post-translationally modified [2], [66], to interact with other factors [67], [68], [69], or to undergo conformational changes [70] upon temperature shift. Thermoregulation mechanisms controlled by DNA-binding proteins or by DNA-supercoiling status are unlikely to be involved in capsule thermoregulation in *S. pyogenes* since capsule thermoregulation occurs at a post-transcriptional level. Also, a typical riboswitch does not appear to govern capsule thermoregulation in *S. pyogenes* since capsule thermoregulation exhibits higher quantities of capsule at lower temperature. It appears likely that capsule thermoregulation in *S. pyogenes* involves a novel mechanism that has not yet been explored.

The expression of *S. pyogenes* pili is also thermoregulated; they are expressed more at sub-body temperature (30°C) than body temperature (37°C) [71]. The pili are also adhesin molecules binding both human tonsil epithelium and primary human keratinocytes [72]. Since both capsule and pili are adhesins and expressed more at sub-body temperature, these certainly indicate the benefit of thermoregulation of these adhesins for binding and subsequent penetration. The underlying mechanism of pili thermoregulation has not yet been studied but seems to be different from that of capsule thermoregulation. Unlike capsule thermoregulation, pili thermoregulation occurs at the transcriptional level [71]. This pili thermoregulation appears to not be mediated by the CovRS two-component system because the expression level of the pili biogenesis genes in the *cpa* operon is the same between the wild type and a CovR mutant [30].

CvfA was discovered during transposon mutagenesis experiments screening for changes in capsule thermoregulation. Our previous study showed that CvfA, a putative endoribonuclease, also influences the expression of metabolic and virulence genes depending on growth phase and nutritional stress, and that influence is independent of the ppGpp-associated stringent response [17]. In chemically defined media, CvfA represses the expression of the genes of M protein (*emm*), streptokinase (*ska*), and the CAMP factor (*cfa*) in carbohydrate deficient conditions, and activates the expression of the gene of SpeB (*speB*) in peptide deficient conditions. Recent studies of the CvfA ortholog in *Bacillus subtilis*, RNaseY, indicate that the protein is an endoribonuclease that interacts with glycolytic enzymes (enolase and phosphofructokinase), ribonucleases (PNPase and RNase J1), and an RNA helicase (CshA) [73], [74], [75]. We also demonstrated that CvfA interacts with the glycolytic enzyme enolase in *S. pyogenes* [17]. Since the RNA degradosome in *E. coli* is composed of components (enolase, RNase E, PNPase, and an RNA helicase, RhlB) similar to the proteins CvfA interacts with [73], CvfA appears to be a component of the RNA degradosome of bacteria in the phylum of firmicutes including *S. pyogenes*, *S. aureus*, and *B. subtilis* [73], [74], [75].

It appears that CvfA is not involved in the degradation of the capsule mRNA since the CovR^−^CvfA^−^ strain produced almost the same amount of capsule transcript as the CovR^−^ strain at both 25°C and 37°C. Thus, CvfA might affect the expression of a regulator controlling capsule thermoregulation. In some cases, the influence of CvfA on virulence gene expression is mediated by regulators. For example, the production of the major secreted protease SpeB is greatly reduced in the CvfA null strain, and this reduction is restored to the wild type level when the major transcriptional activator gene for *speB* expression, *ropB*, is additionally expressed *in trans* in the CvfA null strain [17]. This result indicates that CvfA influences the production of SpeB through the transcriptional regulator RopB. Similarly, CvfA of *Staphylococcus aureus* influences the expression of alpha-hemolysin through SarZ, a positive transcriptional regulator [76]. Once the transcriptional repression by CovR is derepressed, capsule genes are transcribed at the same rate regardless of temperature, while production of HasA is apparently de-repressed below 37°C. This suggests that a repressor system for the translation of the capsule gene transcript controls capsule thermoregulation and that CvfA might influence the degradation of the repressor transcript depending on environmental temperature. Our recent transposon mutagenesis discovered two new chromosomal loci that influence capsule thermoregulation. These transposon-generated mutants showed the same phenotype as the transposon-generated CvfA^−^ mutant; they overproduced capsule regardless of incubation temperature. One capsule overproducer has a transposon insertion at 187 nucleotides upstream of the *hasA* start codon (110 nucleotides upstream of the −35 promoter element). It appears that the region around the insertion site is not involved in transcriptional regulation of capsule gene expression because the transposon insertion site is far upstream of the capsule promoter and capsule thermoregulation is controlled at the translational level. Also, the region is not part of either the transcript of or transcriptional regulation site for *SPy_2199*, the divergently transcribed immediate upstream gene of capsule genes, because the disruption of *SPy_2199* does not influence capsule thermoregulation (Table 1). Thus, even though the transposon-inserted region is not a part of the capsule gene transcript or a transcriptional regulation site, the region is necessary for capsule thermoregulation. This region does not encode an open reading frame even for a small peptide, so it might encode a small non-coding RNA. One possibility of CvfA's role in capsule thermoregulation is through the regulation of this putative small non-coding RNA expression. The other capsule overproducer had a transposon insertion in a prophage gene. HSC5 has three prophages on its chromosome. The prophage in which the transposon inserted resides between the genes of excinuclease ABC subunit A (the ortholog of Spy_1825) and putative divalent cation transport protein (the ortholog of Spy_1827). The prophage gene in which the transposon inserted is the ortholog of *SpyM3_1254*. Thirty nine open reading frames exist downstream of *SpyM3_1254* in the same direction, so *SPyM3_1254* or a downstream gene or genes could be responsible for the phenotype caused by the transposon insertion (polar effect). Hence, one of those genes may produce a regulator required for capsule thermoregulation, and CvfA may influence the expression of the regulator.
